# Ultrasound and Magnetic Resonance Imaging of Burned-Out Testicular Tumours: The Diagnostic Keys Based on 48 Cases

**DOI:** 10.3390/cancers14164013

**Published:** 2022-08-19

**Authors:** Thomas Desmousseaux, Emmanuel Arama, Florian Maxwell, Sophie Ferlicot, Chahinez Hani, Karim Fizazi, Cédric Lebacle, Yohann Loriot, Meriem Boumerzoug, Julian Cohen, Nada Garrouche, Laurence Rocher

**Affiliations:** 1 Service de Radiologie, APHP Hôpitaux Paris Saclay, Hôpital Antoine Béclère, 157 Rue de la Porte de Trivaux, 92140 Clamart, France; 2 Service de Radiologie, APHP Hôpitaux Paris Saclay, Hôpital Bicêtre, 78 Avenue du Général Leclerc, 94270 Le Kremlin-Bicêtre, France; 3 Faculté de Médecine, Université Paris Saclay, 63 Rue Gabriel Péri, 94270 Le Kremlin-Bicêtre, France; 4 Service d’Anatomo-Pathologie, APHP Hôpitaux Paris Saclay, Hôpital Bicêtre, 78 Avenue du Général Leclerc, 94270 Le Kremlin-Bicêtre, France; 5 Service d’Oncologie Médicale, Institut Gustave Roussy, Université Paris-Saclay, 94805 Villejuif, France; 6 Service de Chirurgie Urologique, APHP Hôpitaux Paris Saclay, Hôpital Bicêtre, 78 Avenue du Général Leclerc, 94270 Le Kremlin-Bicêtre, France; 7 Laboratoire d’Imagerie Biomédicale Multimodale Paris Saclay BioMaps, IR4M, UMR8081, 4, Place du Général Leclerc, 91401 Orsay, France

**Keywords:** testicular neoplasms, burned-out tumour, neoplasm regression, multiparametric magnetic resonance imaging, ultrasonography, multiparametric ultrasound

## Abstract

**Simple Summary:**

In rare cases, testicular cancers regress spontaneously. This is known as a burned-out testicular tumour. Few data have been published regarding the radiological appearance of these lesions. In this study, we describe the lesions observed in 48 patients using several imaging techniques (conventional ultrasound, advanced ultrasound techniques and MRI). In both ultrasound and MRI, there are features that allow the recognition of such lesions and may help in the correct management of these patients by proposing orchiectomy.

**Abstract:**

The spontaneous regression of testicular germ-cell tumours is a rare event whose mechanisms have yet to be elucidated. In the majority of published cases, tumour regression is concomitant with the metastatic development of the disease. Residual lesions, often referred to as burned-out testicular tumours (BOTTs), are difficult to diagnose due to the paucity of published data, especially in the field of imaging. The aim of this article is to describe the radiological signs of BOTTs on multimodal ultrasound and multiparametric MRI from a series of 48 patients whose diagnosis was confirmed histologically. The demographic, clinical and laboratory characteristics of the patients are studied, as well as the data of the imaging examinations, including conventional scrotal ultrasound, shear-wave elastography, contrast-enhanced ultrasound (CEUS) and multiparametric MRI. A total of 27 out of 48 patients were referred for investigation of primary testicular lesion following the discovery of retroperitoneal metastases, 18/48 patients were referred because of lesions suspected on an ultrasound that was performed for an infertility work-up, and 3/48 were referred because of scrotal clinical signs. Of these last 21 patients (infertility work-up/scrotal clinical sign), 6 were found to be metastatic on the extension work-up. Of the 48 orchiectomy specimens, tumour involution was complete in 41 cases, and a small active contingent remained in 7 cases, with 6 suspected upon advanced US and MRI. Typically, BOTTs appear on a conventional ultrasound as ill-delineated, hypoechoic and hypovascular nodular areas. Clustered microliths (60.4%) and macrocalcifications (35.4%) were frequent. Shear-wave elastography showed areas of focal induration (13.5 ± 8.4 vs. 2.7 ± 1.2 kPa for normal parenchyma, *p* < 0.01) in 92.5% of the patients for whom it was performed, and contrast ultrasonography demonstrated hypoperfusion of these lesions. Of the 42 MRIs performed, BOTTs corresponded to nodules on T2-weighted sequences (hyposignal) with significantly increased ADC values compared with healthy parenchyma (2 ± 0.3 versus 1.3 ± 0.3 × 10^−3^ mm^2^/s, *p* < 0.01) and an enhancement defect after injection. This enhancement defect overlapped the lesions visible on T2-weighted sequences in most cases. In the case of predominant partial regression, an enhanced portion after contrast injection was visible on MRI in all seven patients of our series, and in six of them a focal diffusion restriction zone was also present. Spontaneously involuted testicular germ-cell tumours have specific radiological signs, and all of the mentioned examinations contribute to this difficult diagnosis, even histologically, because there is no tumour cell left. These signs are similar whether the patient is initially symptomatic metastatic or whether the discovery is fortuitous on the occasion of an infertility work-up, and whatever the seminomatous or non-seminomatous nature of the germ-cell tumour, when this can be determined. The appearance of regressed germ-cell tumours is often trivialized, which can lead to the wrong diagnosis of an extra gonadal germ-cell tumour (in metastatic patients) or of scarring from an acute event such as trauma or infection, which is not recognized or forgotten. In our series, two patients had an unrecognized diagnosis in their history, with local and/or distant recurrence. An improvement in diagnosing burned-out tumours, combining advanced US and MRI, is necessary in order to optimize patient management, with special attention paid to asymptomatic patients, to prompt extension screening and orchiectomy with analysis of the whole testis. This may reveal a persistent viable tumour or lesions of germinal neoplasia in situ, which are precursors of testicular germ-cell tumours.

## 1. Introduction

Testicular cancer is a rare condition but is the leading cause of malignancy in young men [1] and its incidence is expected to increase in the coming years [2]. More than 90% of these cancers are germ-cell tumours with an approximately equal proportion of seminomatous germ-cell tumours (SGCTs) and non-seminomatous germ-cell tumours (NSGCTs). A rare development is the spontaneous regression of the primary testicular tumour, resulting in its partial or complete replacement by a patch of fibrosis. Such lesions have been commonly described as burned-out testicular tumours (BOTTs) and are commonly diagnosed in symptomatic metastatic patients. In 2016, this entity was included in the latest WHO classification of tumours of the urinary tract and male genital organs, with the ICD-O code 9080 and behaviour categorized as 1, meaning unspecified, borderline or uncertain behaviour [3]. Recent studies have incriminated BOTTs to be responsive of Kelch-like protein-11 encephalitis [4].

Scrotal ultrasound is the reference examination for testicular exploration [5] because of its accessibility and the location of the testis, which allows the use of high-frequency probes whose spatial resolution is unmatched by other imaging modalities. In some cases, the results do not allow a satisfactory characterization [5,6,7], and advanced ultrasound techniques such as shear-wave elastography (SWE) [8,9] and contrast-enhanced ultrasound (CEUS) [10] could help to distinguish between benign and malignant lesions. MRI is an efficient supplemental imaging modality for scrotal pathology [11,12,13,14] and is recommended as a second-line exam to characterize testicular lesions with equivocal ultrasound findings [15]. Making the diagnosis of a burned-out tumour leads to the suggestion of an orchidectomy, in the aim to prevent local recurrence, and perhaps also metastatic lesions or recurrences. In the case of metastatic status, the treatment will include chemotherapy with a short delay. Rarely, for SGCT, radiotherapy will be suggested for limited retroperitoneal nodes.

The aim of this study was to assess the multiparametric ultrasound and MRI characteristics of burned-out testicular tumours based on findings in 48 patients.

## 2. Materials and Methods

### 2.1. Case Selection

This study was approved by the French Ethics Committee for Research in Medical Imaging (CERIM) review board (CRM-2107-17).

In this two-centre study, from January 2010 to March 2022, cases of presumed burned-out testicular tumours were collected from the scrotal ultrasonography databases of the Departments of Radiology of Béclère and Bicêtre University Hospitals, Paris, France. Both departments are specialized in male urogenital imaging. Patients were mainly referred to US scrotal examinations for symptomatic metastatic status or infertility screening. Patients with a suspected BOTT on imaging but who did not undergo surgery, including orchiectomy, were excluded. Similarly, patients who underwent orchiectomy but whose diagnosis was not histologically confirmed were excluded. Ten patients with a BOTT revealed during investigations for infertility, previously published as a case series, were included in this study [16]. We decided to include patients for whom the shrinking process was radiologically and pathologically mostly dominant, with a very tiny part of the active tumour.

For each case, patient records were retrospectively reviewed, and demographic features, clinical symptoms prompting initial imaging, histopathology reports, and follow-up studies were collected. Tumour-marker values at diagnosis including human chorionic gonadotropin (HCG), alpha-fetoprotein (AFP), and lactate dehydrogenase (LDH) were collected.

### 2.2. Imaging

#### 2.2.1. Ultrasound Examination

Scrotal US was performed by four radiologists (LR, FM, EA, NG) with expertise in urogenital imaging (respectively, 25, 5, 3 and 3 years of experience) and who were qualified in the use of SWE and CEUS. US was performed using either an Aplio™ US device (Toshiba Medical Systems Europe, Zoetermeer, The Netherlands) with linear broadband transducers (PLT-805 AT or PLT-1204 BT) or an Aixplorer™ US diagnostic device (Supersonic Imagine, Aix-en-Provence, France) with broadband linear transducers (SL 15-4 or SL10-2). The transducer choice depended on the lesion depth. The colour Doppler settings were optimized for slow flow detection (e.g., velocity ranging from 3.5 to 5 cm/s) on both systems. Ultrasensitive Doppler was performed when available.

Still frames and cine loops were systematically archived on our Picture Archiving and Communication System (PACS) for review as well as standardized US reports including: testicular volume (in millilitres, obtained by multiplying the three diameters by 0.523); echotexture; presence and description of nodules (location, number, size, echogenicity, margins, vascularization, stiffness as assessed with SWE if available); presence and grading of microliths and macrocalcifications; CEUS results if performed; presence and grading of varicocele; evaluation of the epididymis and vas deferens; presence or absence of a hydrocele. The PACS and standardized US reports used were the same in both radiology departments.

For each patient, the testis volume, the presence, distribution and grading of microliths (<5 per field of view (FoV); >5 per FoV; diffuse) [17] as well as macrocalcifications (calcification ≥ 3 mm), and the number (1; 2; >2), size (of the largest if multiples), shape, echogenicity (hypoechoic; isoechoic; hyperechoic) and vascularity (hypovascular; isovascular; hypervascular pattern)—as assessed with colour Doppler US (CDUS)—of the lesions were collected from standardized US reports and visualizations of the recorded data.

Real-time SWE was performed using the Aixplorer™ ultrasound diagnostic system with an elasticity scale of 20 to 30 kPa. A slow scan of the entire pathological testis and the local stiffness was measured using a region of interest placed on the hardest part of the pathological area and on the normal parenchyma adjacent to the lesion or on the contralateral testis. At least 3 consecutive measurements were performed, and the mean value was calculated for each region of interest.

CEUS was performed on the Aplio™ system or Aixplorer™ system using continuous low mechanical index contrast-harmonic imaging, after a peripheral intravenous bolus injection of 4.8 mL of SonoVue™ (Bracco Spa, Milan, Italy) followed by a 10 mL bolus injection of a saline solution. Continuous observation of the lesion was performed until the microbubbles disappeared, and was recorded as cine loops. We assessed the perfusion pattern.

#### 2.2.2. MRI Examination

MRI explorations were initially performed on a 1.5 Tesla Philips Healthcare Achieva™ system (11/42) (Best, The Netherlands), a 1.5 Tesla Siemens Healthcare Magnetom Aera™ system (21/42) or a 3 Tesla Siemens Healthcare Skyra™ system (10/42) (Erlangen, Germany). The imaging protocol included axial, coronal, and sagittal T2 turbo spin echo, axial T1 turbo spin echo, diffusion MRI with two B-values (0–800 for the first exams, 50–1000 for the next exams), and dynamic T1 contrast-enhanced sequences that were acquired in the axial plane. We collected the following data: T2WI lesion shape (round or oval-shaped nodule versus focal area with irregular boundaries); number of lesions on T2WI (1; 2; >2); T2WI, T1WI and DWI signal (hyposignal; isosignal; hypersignal); apparent diffusion coefficient (ADC) values in the ADC map of the lesion and the normal testis parenchyma using the manual region of interest; enhancement of the nodule compared with that of the normal contralateral parenchyma (hypoperfusion; isoperfusion; hyperperfusion); time–signal-intensity curve type (15) (if available); and the size of the hypo- or hyperperfused area compared to the T2WI lesion, after intravenous injection of gadolinium chelates (Dotarem™, gadoteric acid, 0.2 mL per kg body weight, Guerbet SA, Aulnay-sous-Bois, France).

#### 2.2.3. Complementary Examinations

In the case of metastatic disease, the presence and localization of metastases were collected according to contrast-enhanced computed tomography (CT) results.

### 2.3. Surgical Procedure and Pathological Analysis

Each patient was surgically managed with a complete orchiectomy 15 to 31 days after imaging. Pathological analysis was performed by a senior pathologist (SF). Immunohistochemistry staining was performed on paraffin-embedded tissues using placental alkaline phosphatase (PLAP) antibody (Dako, dilution 1:150) for all cases. Data were retrospectively collected from the histology reports. In the case of metastasis, CT-guided or surgical biopsy was performed followed by pathological analysis, and the results were collected from the histology reports.

### 2.4. Statistical Analysis

Paired continuous variables (i.e., stiffness (kPa) and ADC values) were compared using the paired Student’s *t*-test. The Wilcoxon–Mann–Whitney test was used to compare continuous variables (e.g., lesion size) and Fisher’s exact for non-continuous variables (e.g., presence of macrocalcifications). Statistical analyses were carried out using R statistical software (Version 4.1.0).

## 3. Results

**Figure 1:**
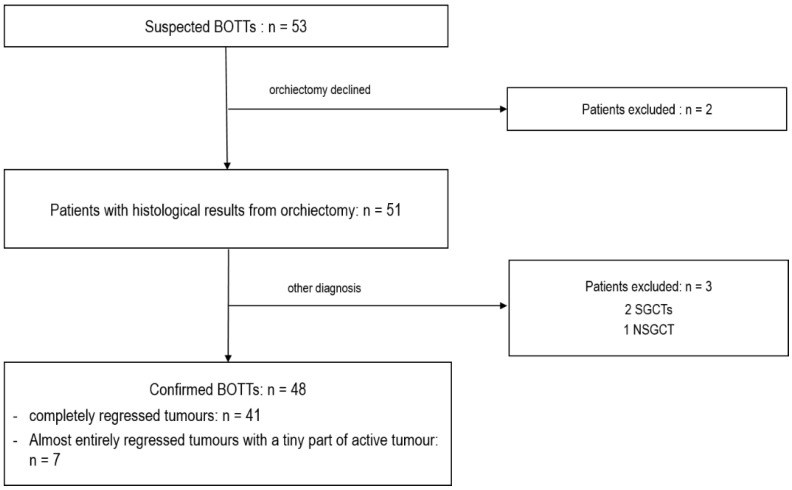
Flow chart of patient selection.

Fifty-three cases of a suspected BOTT were found in the databases among the 17,011 scrotal US examinations performed in the departments between January 2010 and March 2022. Two patients were excluded from the study because they declined the recommended orchiectomy. The remaining 51 patients underwent complete orchiectomy. For three patients, the lesion suspected to be a BOTT on US corresponded to seminomas (2/3) or NSGCT (1/3) without histological evidence of tumour regression, and these were therefore excluded. Thus, a total of 48 patients were included with histologically confirmed BOTT diagnosis. Among these, 41 cases of complete regression were observed, and in 7 cases a small viable tumour nodule was found among large areas of tumour regression, corresponding to partial regression (Figure 1).

### 3.1. Demographic, Clinical and Laboratory Data 

Demographic, clinical and biological data are summarized in Table 1.

Patient ages ranged from 20 to 68 years, with a median age of 39 years (IQR: 32–43).

Twenty-seven patients (56.3%) were initially referred for testicular exploration, after the discovery of metastatic lesions, in order to search for a primary testicular tumour. Eighteen patients (37.5%) were initially referred for infertility assessment, one for abnormal self-examination, one for testicular pain and one for a second opinion after the incidental discovery of a testicular lesion in an infectious context.

All patients diagnosed with completely regressed testicular tumours were considered not to have suspected testicular cancer on clinical examination. Two of the six patients diagnosed with partially regressed testicular tumours presented with an abnormal scrotal clinical examination: one had a palpable indurated nodule (patient referred for abnormal self-examination) and the other complained of scrotal pain. Thus, a total of 46 patients (95.8%) had no clinical evidence of testicular cancer. For one patient with an abnormal initial examination made by the general practician, and who underwent an initial US revealing a supracentimetric hypoechoic vascularized lesion, which was clearly visible on the pictures, but the patient was anxious to consult a urologist. Finally, two months after, the palpation of the urologist revealed a small testis (US/MRI suggested a BOT). This was the only case with a proven “shrinking” process, and it remained at 2 mm of active SGCT upon histological examination.

Metastases were found in 32 patients (66.7%), including 6 totally asymptomatic patients referred for investigation in the context of infertility. Pathology revealed 22 cases of SGCT metastasis and 10 of NSGCT metastasis. In one patient, a single unbiopsied retroperitoneal lymph node was considered to be an SGCT metastasis, with the testis histological examination revealing a partially regressed seminoma. Retroperitoneal nodes or masses were found in all metastatic patients. Eight patients, who were finally diagnosed with NSGCTs, also had other metastatic locations: bones (5/8); lung (5/8); liver (2/8); brain (1/8); supra-clavicular node (1/8); mesenteric mass (1/8).

The average volume of the testis containing the BOTT was 7.9 ± 3.4 mL, whereas the average volume of the contralateral testis was 11.4 ± 5.5 mL (significant difference, *p* < 0.01).

Tumour markers (human chorionic gonadotropin and alpha-fetoprotein) were available for 42 patients (38 with complete regression of the testicular tumours and 4 with partial regression). Elevated hCG values were found in 11 patients: 6 with histologically proven metastases of NSGCTs (mean value: 135,149 ng/mL) and 5 with seminoma metastasis (mean value: 18 ng/mL). Elevated AFP values were found in four patients with metastasis of NSGCTs (mean value: 13,685 ng/mL). In the seven patients with partially regressed tumours (one of which was metastatic), no tumour-marker elevation was found. No elevations were found in the 11 non-metastatic patients for whom data were available.

### 3.2. Imaging Findings

All patients underwent a conventional scrotal ultrasound. SWE was performed in 40 patients, CEUS in 25 patients and multiparametric scrotal MRI in 42 patients. A total of 25 patients underwent all imaging modalities.

BOTTs were in the right testis in 23 patients and in the left testis in 25 patients.

#### 3.2.1. Conventional US Findings 

**Figure 2:**
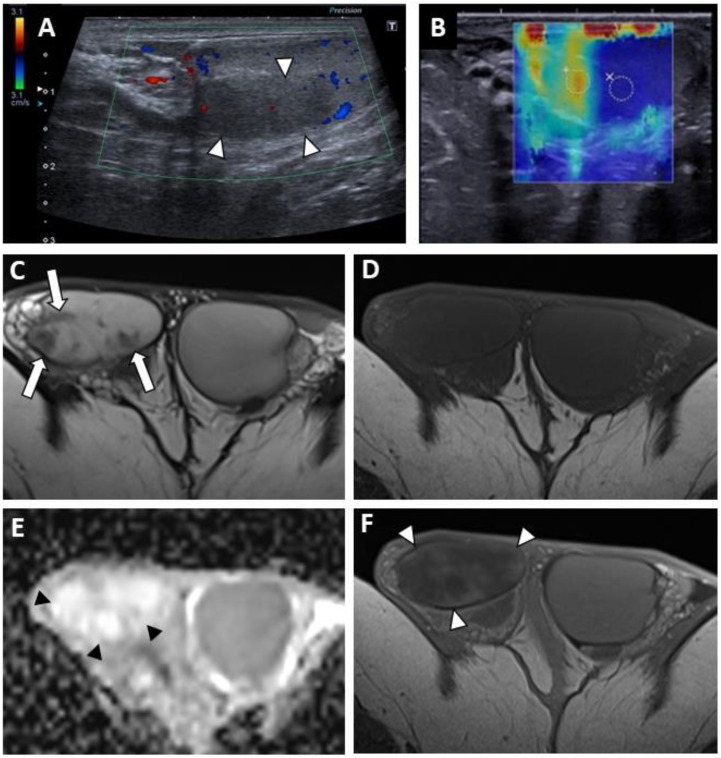
BOTT of the right testis in a 40-year-old man (symptomatic retroperitoneal nodes). Longitudinal image of US on Colour Doppler mode showing poorly circumscribed hypoechoic and hypovascularised nodular areas (white arrowheads) (**A**). Shear-wave elastography mapping showed a focal induration (**B**) (different slice plane from image (**A**)). Axial T2W image showed multiple irregular areas with a hypo-intense signal (white arrows) (**C**). Axial T1W image without a detectable focal lesion (**D**). ADC map of the diffusion sequence demonstrated a focal area with an elevated ADC value compared with the contralateral testis (black arrowheads) (**E**). After the intravenous injection of gadolinium chelate, confluent areas with reduced enhancement were visible (note that these areas are overlapping the nodules visualized in T2) (**F**).

**Figure 3:**
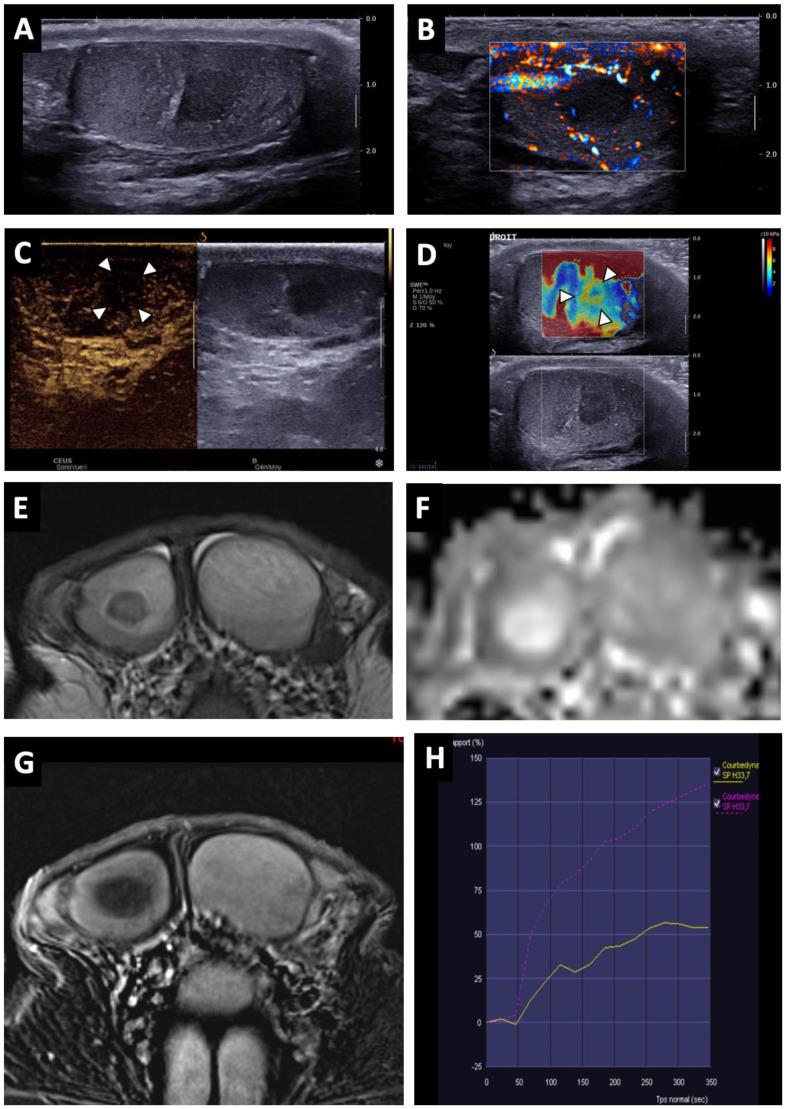
BOTT of the right testis in a 41-year-old man (infertility screening). Transverse B-mode US showed an ill-delineated hypoechoic nodular area (**A**). With colour Doppler US, the hypoechoic nodule was frankly hypovascular upon Ultrasensitive Doppler (**B**). CEUS demonstrated a hypo-enhanced (white arrowheads) area in concordance with the B-mode nodule (note that even the adjacent testis parenchyma was also poorly enhanced) (**C**). SWE map showed an area of increased stiffness in concordance with B-mode nodule (**D**). MRI axial T2W image showing a nodule with a hypo-intense signal (white arrow) (**E**). ADC map of the diffusion sequence demonstrated a focal area with an elevated ADC value compared with the contralateral testis (**F**). After the intravenous injection of gadolinium chelate, an area of reduced enhancement of the nodule was visible (**G**). Nodule enhancement followed the yellow time–signal-intensity curve (type 1) and the contralateral parenchyma corresponded to the purple one (**H**).

Lesions appeared as hypoechoic nodular areas (Figure 2 and Figure 3) for 44 patients (91.7%) and an entire hypoechoic testis infiltration for 4 (8.3%). The lesion was singular in 36 patients (75%), there were 2 nodules in 3 patients (6.3%) and more than 2 in 9 patients (18.8%). Conventional US findings results are summarized in Table 2.

All lesions had ill-delineated margins. In 42 cases (87.5%), the lesions appeared hypovascular compared to normal parenchyma (Figure 2 and Figure 3). In six patients, a hypervascular nodule with sharp boundaries was found within (four cases) or peripheral to (two cases) a hypovascular nodular area using CDUS, and corresponded to residual seminomas upon pathological analysis. One patient, who was finally diagnosed with a partially regressed seminoma, had no focal US abnormality that was suggestive of a viable tumour.

The mean diameter of the measurable lesions (i.e., excluding cases with entire testis infiltration) was 13.1 ± 4.5 mm without a significant difference between patients with seminomas and patients with NSGCTs.

Clustered microliths were observed at the periphery of the lesions in 29 patients (60.4%) and macrocalcifications in 17 patients (35.4%). Microlithiasis (i.e., MLs with a random distribution in opposition to clustered MLs) were encountered in 13 patients (grading is specified in Table 2).

There were no statistically significant differences in US lesion characteristics between patients with NSGCTs and SGCTs.

Twenty-five CEUS were performed. A reduced vascularity of the entire testis, which was more pronounced in the hypoechoic region, was visible for 22 patients (Figure 3C). In three cases, corresponding to patients with hypervascular nodules in CDUS, an early and strongly enhanced small nodule was visible, surrounded by an area of reduced enhancement in two cases, and at its periphery in one case. Few microbubbles were observed within the hypovascular lesions in all cases.

SWE exhibited a focal area of increased stiffness (Figure 2B and Figure 3D) corresponding to hypoechoic lesions in 37 of 40 patients (92.5%). In three patients (7.5%) there was no detectable focal stiffness abnormality. Stiffness measurements in 30 patients indicated a mean value of 13.7 ± 8.4 kPa in areas of elevated stiffness versus 2.6 ± 1.3 kPa in normal testicular parenchyma (significant difference, *p* < 0.01). The mean stiffness ratio was 5.3 ± 2.4. No significant difference was found when comparing hypervascular nodules and the hypovascular surrounding areas in the case of partial tumour regression.

#### 3.2.2. MRI Findings 

MRI findings are summarized in Table 3.

**Figure 4:**
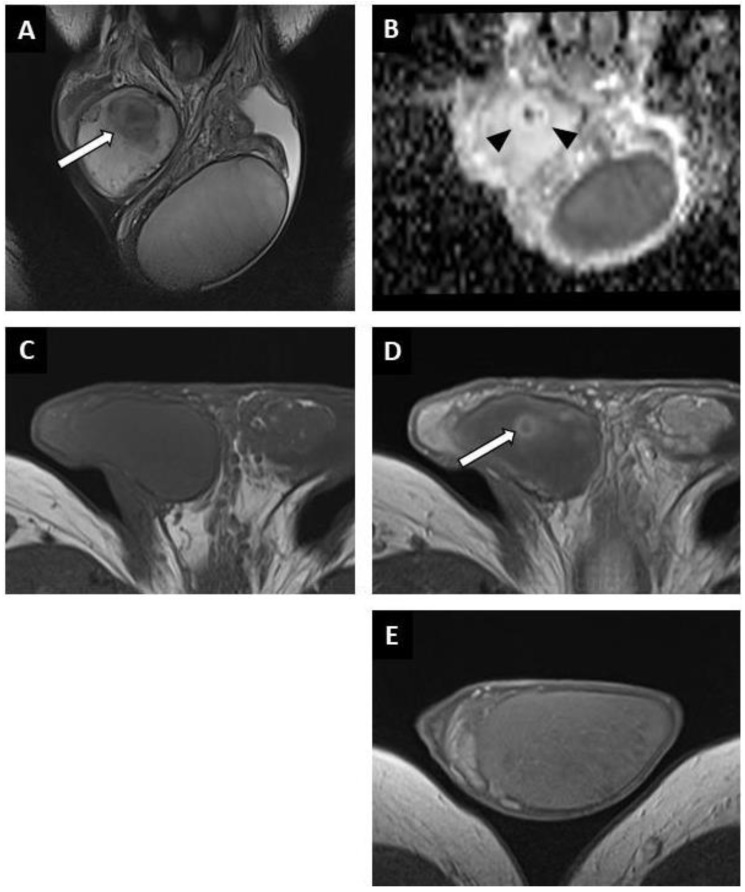
Partially regressed seminoma of the right testis in a 52-year-old man (symptomatic metastatic retroperitoneal nodes). Coronal T2W image showing a nodule with ill-delineated margins in hyposignal (white arrow) (**A**). Coronal reconstruction of an ADC map showing a focal elevation of ADC values (black arrowheads) with a central area of restriction (in black) (**B**). Before injection, no focal abnormality was visible on the axial T1W sequence (**C**). After intravenous injection of the contrast agent, a large area of reduced enhancement was visible (overlapping the lesions compared to the T2W images) with a central enhanced nodule (white arrow) (**D**). Axial post-injection T1W image of the left testis showing the usual testicular parenchymal enhancement (**E**). Pathological analysis revealed a 5 mm residual seminoma within a 2 cm fibro-hyaline patch.

BOTTs appeared as round or oval nodules (81%) (Figure 3 and Figure 4) or a focal area with irregular boundaries (19%) on the T2 sequences (hyposignal). No lesions were visible on the T1 sequences (isosignal) (Figure 4C).

A singular lesion was found in 31 patients (73.8%), 2 in 4 patients (9.5%) and multiple lesions (at least 3) in 7 cases (16.7%). The number of lesions visible on MRI was the same as on US in 38 patients. In two cases an additional lesion was found on MRI and in two cases a lesion that was visible on ultrasound was not visible on MRI. In patients with entire testis infiltration on US, MRI revealed multiple nodules or focal areas with irregular boundaries.

The diffusion-weighted sequences showed nodular areas of reduced signal with increased ADC values (mean value: 2 ± 0.3 × 10^−3^ mm^2^/s) (Figure 2E and Figure 3F) compared to contralateral parenchyma (mean value: 1.3 ± 0.3 × 10^−3^ mm^2^/s) in all patients (significant difference, *p* < 0.01). The mean ADC ratio was 1.6 ± 0.3. Within these areas with increased ADC values, a restrictive nodule (Figure 4B) was detected in six of the seven patients that were diagnosed with a partially regressed testicular tumour.

Dynamic contrast-enhanced sequences showed areas of reduced enhancement in all cases. The size of this area matched that of the lesion on the T2 sequences (Figure 3G) in 11 patients (26.2%), and in 31 patients (73.8%) it overlapped the lesion with reduced enhancement extending to the peripheral parenchyma (Figure 2F and Figure 4D). In the cases of partial tumour regression, dynamic contrast-enhanced sequences revealed early and strongly enhanced focal abnormalities within areas of reduced enhancement, corresponding to nodules in six patients (Figure 4D), and an ill-delineated area with increased enhancement in one (corresponding to the patient in whom no focal abnormality that was suggestive of a viable tumour was found upon US examination).

Time–signal-intensity curves were created (Figure 3H) for 23 patients with complete tumour regression, showing type 0 curves in 7 cases and type 1 in 16 cases. Type 2 (two patients) or 3 (five patients) time–signal-intensity curves were observed in focal lesions corresponding to viable seminoma.

There were no significant differences in the MRI lesion characteristics between patients with NSGCTs and SGCTs.

### 3.3. Pathological Findings

**Figure 5:**
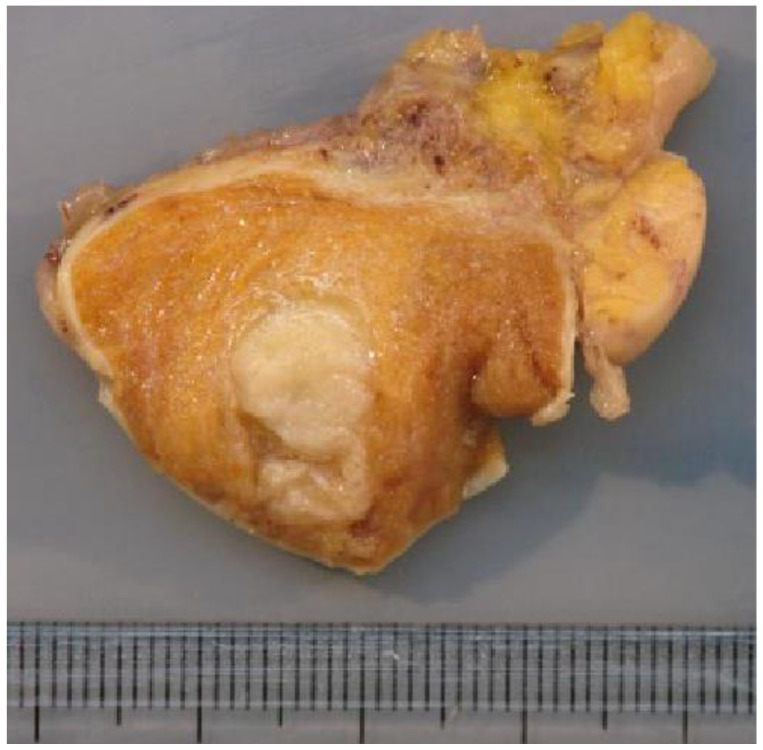
Macroscopic examination showing a firm whitish nodule corresponding to the BOTT (same patient as Figure 3).

The macroscopic examination showed firm white nodules (Figure 5) in 45 cases, with a mean diameter of 17 mm (ranging from 6 to 40 mm). The histological examination revealed nodular fibrous scars with dense collagenous tissues and remnants of hyalinized seminiferous tubules in these patients. In three patients, no nodular lesion was found and a multinodular pattern of hyalinized seminiferous tubules was found upon microscopic examination.

A lymphoplasmacytic infiltrate in the fibrous tissue was reported in 28 cases (58%), was absent in one and was not specified in the others. In all cases there were completely hyalinized tubules that were mainly associated with a “Sertoli cell only” pattern peripheral to the scar. Impaired spermatogenesis was seen in all the patients. Intratubular germ-cell neoplasia (IGCN) was found in nine patients (18.8%) peripheral to the area of scarring, which was associated with partial tumour regression in four cases. In the six cases of partial tumour regression, the lesions found were pure seminomas.

## 4. Discussion

A burned-out testicular tumour is defined as the spontaneous regression of a primary testicular germ-cell tumour. This phenomenon was first reported by Prym in 1927 [18]. Then, in 1961, Azzopardi et al. [19] defined the concept of a burned-out testicular tumour and published the histological features of such lesions. The radiological diagnosis of this type of tumour is difficult, especially as there are few published studies that focus on the imaging of burned-out testicular tumours. The studies with the largest numbers of patients were mainly interested in the clinical and histological features [20,21].

To our knowledge, our series is the largest that reports the radiological characteristics of BOTTs, and one of the few to consider all of the modalities available in the current practice to date.

In most of the published cases, BOTTs are discovered during the search for a primary tumour in a metastatic patient [20,21,22]. In our study, only 56.3% of patients were referred for this reason, and 37.5% for infertility. This is probably related to a recruitment bias as many infertile men are screened in our radiology departments. Infertile men have an increased risk of developing testicular cancer, particularly SGCTs [1,23,24]. This might explain the over-representation of seminomas in our study. In all patients with completely regressed testicular tumours, the clinical examination of the testis was considered non-suspicious, and the only abnormality was testicular atrophy. The association of retroperitoneal nodes or masses and testicular atrophy should raise the suspicion of a BOTT.

Seven cases of partial tumour regression are reported in this study. Testicular cancer was clinically suspected in only two of them. Distinguishing between a regressing testicular cancer and the development of an active lesion from a BOTT is difficult. The clinical course may help if the patient describes a recent loss of volume or conversely a recent increase in testicular volume (especially if the testicle was initially small). The clinical history of our patients suggested that these were cases of incomplete regression rather than recurrence as none of them had a known pre-existing scrotal abnormality, and one patient had a rapid decrease in the volume of the testis in which the BOTT was found. However, we believe that recurrence from a BOTT is possible, as we have followed two patients in the department with testicular lesions that were highly suggestive of BOTT but who refused surgery, and in whom suspicious nodules that were later confirmed as seminomas appeared.

Tumour markers do not seem to help in the diagnosis of a BOTT as they were normal in all non-metastatic patients and in all asymptomatic metastatic patients.

We have chosen to present cases of partially regressed testicular tumours among the completely regressed tumours because of their common features on US and MRI exploration, as well as on histological analysis. Their radiological diagnosis is less difficult due to the presence of a hypervascular nodular portion, which is usually found in cases of viable testicular cancer. The diagnosis of a completely regressed tumour is more challenging, particularly on US exploration. In our study, the typical ultrasound appearance of a BOTT was a hypoechoic, poorly delineated, hypovascular nodular area. A single lesion was found in 36 patients (75%), and multiple lesions (more than two) in 9 patients (18.8%), including 4 cases in which the appearance was a global nodular infiltration of the testis.

Clustered microliths and macrocalcifications were frequently found (60.4% and 35.4% of cases, respectively) in peripheral areas and should raise the suspicious of malignancy [25,26].

Conventional US findings may be insufficient to prompt orchiectomy because BOTTs can be confused with benign lesions, as most testicular avascular or hypovascular lesions are benign [27,28], including segmental testicular infarction [29], epidermoid cysts and hematomas. Indeed, in our study, scrotal US had already been performed in 12 patients outside our radiology departments, but the abnormalities had been considered sequelae without past or present suspicion of testicular cancer. Advanced US techniques such as SWE and CEUS, as well as MRI, can be helpful in characterizing these lesions.

CEUS demonstrated the reduced vascularity of the BOTTs in all cases. Microbubbles were visible within the lesions in all cases, indicating the presence of blood vessels, as US contrast agents are exclusively intravascular. This is consistent with the pathological analysis describing sparse blood vessels in BOTTs [20] and differentiates them from segmental testicular infarction. In 88% of patients, the reduced vascularity of the entire testis and not just the nodular area was visible, which is consistent with the appearance on MRI. CEUS could be useful if MRI is not readily available or accessible by providing an additional argument in favour of a BOTT.

SWE demonstrated focal areas of increased stiffness in 92.5% of patients (13.7 ± 8.4 kPa versus 2.6 ± 1.3 kPa in normal testicular parenchyma (*p* < 0.01)). This appearance is not specific, as increased stiffness has been reported in benign tumours as well as malignant tumours and BOTTs [9,30,31], and a high variability was observed in our study.

Multiparametric MRI demonstrated the presence of a hypovascular nodular area in all cases on dynamic contrast-enhanced sequences. In 73.8% of cases, this hypo-enhanced area overlapped the lesion that was visible on the T2 sequences. The BOTTs exhibited significantly increased ADC values compared to the contralateral parenchyma (2 ± 0.3 × 10^−3^ mm^2^/s versus 1.3 ± 0.3 × 10^−3^ (*p* < 0.01)).

If a BOTT is suspected on conventional US, then the presence of an area of reduced enhancement with elevated ADC values on MRI makes the diagnosis very likely. These elevated ADC values should not be taken as a sign of benignity, even if testicular cancer usually appears as a restrictive lesion on diffusion-weighted sequences (reduced ADC values) [13,32,33].

As the 2016 WHO classification of tumours of the urinary tract and male genital organs indicates, the behaviour of BOTTs is not clearly established. In our series, two patients diagnosed with partially regressed tumours were regularly monitored by US and the viable tumour portion appeared, while the hypoechoic and hypovascular area suggestive of the BOTT had been present for several years. In a study published in 2018, Pedersen et al. reported tumour development in patients who followed up due to testicular macrocalcifications (27). Similarities exist between some patients in our study, and it is possible that the macrocalcifications mentioned are related to BOTTs. In addition, in one patient we excluded because of a histological diagnosis of NSGCT without reported signs of tumour regression, the US—on the basis of which a BOTT was suspected—had been performed two years earlier and clinical abnormalities (including induration of the testis) had appeared in the meantime. IGCN, which are the precursor lesions of testicular germ-cell tumours, were found in nine patients (18.8%) in our study.

Orchiectomy should therefore be considered when such testicular lesions are found on US, especially if scrotal MRI examination confirms the suspicion.

The main limitations of our study are its retrospective design and the small number of patients included, due to the low incidence of the spontaneous regression of testicular germ-cell tumours. In addition, not all patients underwent all imaging modalities or techniques, due to patient or radiology department organizational constraints.

Although ultrasound is an operator-dependent examination, notably by the subjective assessment of certain characteristics such as vascularity, shape, or sharpness of the lesion boundaries, reproducibility bias was minimized by limiting the number of operators and by using a standardized report.

## 5. Conclusions

In young male patients with retroperitoneal nodes, a BOTT should be suspected if a poorly delineated hypoechoic nodular area is seen on US in an atrophied testis. This suspicion is reinforced if microliths or clustered macrocalcifications are seen at the periphery of the lesion.

SWE and CEUS may help to increase confidence in this diagnosis by respectively showing a focal area of increased stiffness and markedly reduced enhancement. In cases of suggestive US, scrotal MRI usually reveals a nodular lesion on T2, with elevated ADC values and reduced enhancement on DCE sequences (usually more extensive than the nodule visible on T2).

Orchiectomy should be considered in the case of such radiological findings as the presence of a BOTT is very likely.

Whole-body CT should be performed to look for metastases and nodes if a lesion suggestive of a BOTT is incidentally found on US in an asymptomatic patient.

## Figures and Tables

**Table 1:** Demographic, clinical, and biological findings.

	SGCTs	NSGCTs	Undetermined	Total
	*n* = 26	*n* = 11	*n* = 11	*n* = 48
Median Age (IQR)	40 (32–47)	34 (27–38)	38 (33–42)	39 (32–43)
**Initial reason for scrotal US**				
- Search of primitive tumour (metastases suspected or proved)	16 (61.5)	11 (100)	0	27 (56.3)
- Infertility	7 (26.9)	0	11 (100)	18 (37.5)
- Abnormal self-examination	1 (3.8)	0	0	1 (2.1)
- Scrotal pain	1 (3.8)	0	0	1 (2.1)
- Suspected male genital infection	1 (3.8)	0	0	1 (2.1)
**Metastatic status**				
- Non-metastatic	5 (19.2)	0	11 (100)	16 (33.3)
- Metastatic	21 (81.8)	11 (100)	0	32 (66.7)
**Metastasis locations**				
- Retroperitoneal node or masse	21 (80)	11 (100)	0	32 (66.7)
- Bone lesion	0	5 (45.4)	0	5 (13.3)
- Lung lesion	0	5 (45.4)	0	5 (13.3)
- Liver lesion	0	2 (18.2)	0	2 (4.2)
- Brain lesion	0	1 (9.1)	0	1 (2.1)
- Supra-clavicular node	0	1 (9.1)	0	1 (2.1)
- Mesenteric mass	0	1 (9.1)	0	1 (2.1)
**Tumoral markers (availability)**	*22*	*11*	9	42
- HCG elevation (mean value (ng/mL))	5 (*18*)	6 (*135,149*)	0	11
- AFP elevation (mean value (ng/mL))	0	4 (*13,685*)	0	4
- LDH elevation (mean value (UI/L))	6 (*822*)	8 (*1198*)	0	14
**Testis histology**				
- Partial regression	7 (26.9)	0	0	7 (14.6)
- Complete regression	19 (73.1)	11 (100)	11 (100)	41 (85.4)

All results are the number of patients (%) unless otherwise mentioned and written in italics. SGCTs: seminomatous germ-cell tumours; NSGCTs: non-seminomatous germ-cell tumours.

**Table 2:** Conventional US findings.

	SGCTs	NSGCTs	Undetermined	Total
	*n* = 26	*n* = 11	*n* = 11	*n* = 48
**Lesion pattern**				
- Nodular area	22 (84.6)	11 (100)	10 (90.9)	44 (91.7)
- Entire testis infiltration	4 (15.4)	0	1 (9.1)	4 (8.3)
**Number of lesions**				
- 1	18 (69.2)	9 (81.8)	9 (81.8)	36 (75)
- 2	2 (7.7)	0	1 (9.1)	3 (6.3)
- >2	6 (23.1)	2 (18.2)	1 (9.1)	9 (18.8)
**Margins**				
- ill-delineated	26 (100)	11 (100)	11 (100)	48 (100)
**Echogenicity**				
- Hypoechoic	25 (100)	11 (100)	11 (100)	48 (100)
Maximal diameter * (SD) (mm)	*13 (4.4)*	*13.5 (5.2)*	*14.1 (4.5)*	*13.1 (4.5)*
**Vascularization**				
- Hypovascular	20 (76.9)	11 (100)	11 (100)	42 (87.5)
- Hypovascular area with hypervascular focal nodule	6 (23.1)	0	0	6 (12.5)
**Microlithiasis** (random MLs)				
- <5 per FoV	3 (11.5)	1 (9.1)	4 (36.4)	8 (16.7)
- >5 per FoV	2 (7.7)	0	0	2 (4.2)
- diffuse	2 (7.7)	0	0	3 (6.3)
- Clustered microliths	15 (57.7)	8 (72.7)	6 (54.5)	29 (60.4)
**Macrocalcifications**	5 (19.2)	5 (45.5)	7 (63.6)	17 (35.4)

All results are the number of patients (%) unless otherwise mentioned and written in italics. SGCTs: seminomatous germ-cell tumours; NSGCTs: non-seminomatous germ-cell tumours. * Largest lesion if multiple.

**Table 3:** MRI findings.

	SGCTs	NSGCTs	Undetermined	Total
	*n* = 22	*n* = 10	*n* = 9	*n* = 41
**Lesion shape (T2WI)**				
- Round or oval nodule	18 (81.8)	8 (80)	9 (100)	34 (82.9)
- Non-nodular area	4 (18.2)	2 (20)	0	7 (17.1)
**Number of lesions (T2WI)**				
- 1	14 (63.6)	9 (90)	7 (77.8)	30 (73.2)
- 2	3 (13.6)	0	1 (11.1)	4 (9.7)
- >2	5 (22.7)	1 (10)	1 (11.1)	7 (17.1)
**Maximal diameter (T2WI)** * (SD) (mm)	*14* (*9.5*)	*12* (*5.9*)	*12.3* (*4.8*)	*13* (*7.6*)
**Lesion signal**				
- Hyposignal on T2WI	22 (100)	10 (100)	9 (100)	41 (100)
- Isosignal on T1WI	22 (100)	10	0	41
- Hyposignal on DWI	17 (77.3)	10	0	32
- Hyposignal with focal hypersignal spot on DWI	5 (22.7)	0	0	5 (12.2)
**ADC mean value (SD)** (×10^−3^ mm^2^/s)				
- Lesion °°	*2 (0.3)*	*1.9 (0.2)*	2.1 (0.3)	*2 (0.3)*
- Normal parenchyma	*1.4 (0.4)*	*1.2 (0.3)*	1.3 (0.2)	*1.3 (0.3)*
**ADC ratio mean value** (SD) (no unit)	*1.6* (*0.3*)	*1.7* (*0.3*)	*1.7* (*0.2*)	*1.6* (*0.3*)
**DCE T1WI**				
- Reduced enhancement matching to lesion	3 (13.6)	3 (30)	3 (33.3)	9 (22)
- Reduced enhancement overlapping the lesion	13 (59.1)	7 (70)	6 (66.7)	26 (63.4)
- Reduced enhancement overlapping the lesion with focal early and strongly enhanced nodule	5 (22.7)	0	0	5 (12.2)
- Reduced enhancement matching to lesion with peripheral ill-delineated area of increased enhancement	1 (4.5)	0	0	1 (2.4)

All results are the number of patients (%) unless otherwise mentioned and written in italics. * Largest lesion if multiple. °° excluding focal restrictive spots if present. SGCTs: seminomatous germ-cell tumours; NSGCTs: non-seminomatous germ-cell tumours.

## Data Availability

Not applicable.
